# Distinct Gut Microbiome Induced by Different Feeding Regimes in Weaned Piglets

**DOI:** 10.3390/genes14010049

**Published:** 2022-12-23

**Authors:** Jie Zhang, Xi Long, Qinfeng Liao, Jie Chai, Tinghuan Zhang, Li Chen, Hang He, Yancong Yuan, Kun Wan, Jinyong Wang, Anfang Liu

**Affiliations:** 1 College of Animal Science and Technology, Southwest University, Chongqing 402460, China; 2 Chongqing Academy of Animal Science, Chongqing 402460, China; 3 College of Animal Science and Technology, Chongqing Three Gorges Vocational College, Chongqing 404155, China

**Keywords:** piglets, breast-fed, formula-fed, gut microbiota

## Abstract

It is well accepted that the gut microbiota of breast-fed (BF) and formula-fed (FF) infants are significantly different. However, there is still a limited number of studies comparing the gut microbiota of BF and FF piglets, despite increasing numbers of FF piglets in the modern pig industry. The present study identified the differences in gut microbiota composition between BF- and FF-weaned Rongchang piglets at 30 days old, using pair-end sequencing on the Illumina HiSeq 2500 platform. The BF piglets had lower microbiota diversities than FF piglets (*p* < 0.05), and the community structures were well clustered as a result of each feeding pattern. Firmicutes and Bacteroidetes represented the most dominant phyla, and *Ruminococcus*, *Prevotella*, and *Gemmiger* were prominent genera in all piglets. *Ruminococcus*, *Prevotella*, *Oscillospira*, *Eubacterium*, *Gemmiger*, *Dorea*, and *Lactobacillus* populations were significantly higher, while *Treponema* and *Coprococcus* were significantly lower in BF piglets compared to FF piglets (*p* < 0.05). The metabolism pathways in the BF piglets were significantly different from FF piglets, which included carbohydrate and amino acid metabolism (*p* < 0.05). In addition, the top 10 abundance of microbiota were more or less significantly associated with the two phenotypes (*p* < 0.05). Collectively, these findings provide probable explanations for the importance of BF in neonates and support a theoretical basis for feeding regimes in indigenous Chinese piglets.

## 1. Introduction

Breast milk is a biofluid produced within the mammary glands of a female mammal and is considered to be the best nutritional source for newborns. Breast milk is rich in numerous nutrients, enzymes, hormones, bioactive molecules, and immune cells which modulate gastrointestinal tract function, the immune system, and brain development, as well as promote healthy growth [1,2]. In addition, breast milk also contains a complex community of bacteria that helps establish the infant’s gut microbiota [3,4]. This not only provides protection against diarrhea morbidity and mortality but also reduces the risk of chronic diseases, such as allergies, diabetes, asthma, inflammatory bowel disease, atopy, and obesity [5,6,7].

Studies have shown that the body’s growth and development are largely determined early in life, and this process is influenced by various factors such as mode of delivery, feeding patterns, gestational age, maternal, environment, time of lactation, and complementary feeding [7,8,9,10]. Among them, the feeding pattern (breast-fed: BF; formula-fed: FF) used is one of the most influential factors [11,12]. In humans, BF infants are leaner than FF infants at one year old [13], and the intestinal crypt depth and mitotic count per crypt were seen to be increased in FF infants compared with BF at 2–6 months old [14]. In rats, small intestine and colon weight was increased in FF compared with BF, alongside higher villous densities, longer villi and deeper crypts, and thicker muscle layers [15,16]. In piglets, the jejunal weight and density were increased in FF neonates compared with BF at 28 days old [17]. Furthermore, Pieper et al. [18] also showed that early response to bovine milk-based compounds in the formula was accompanied by early onset of intestinal functional maturation and impaired barrier function. Based on previous studies, we believed that the formation of gut microbiota would be distinct in BF and FF piglets.

Pigs (*Sus scrofa*) are economically important animals throughout the world. In practice, postpartum sows may provide no, or insufficient milk, and ultra-early weaning makes the piglets unable to receive adequate breast milk, thus causing malnutrition, slow growth, and even death. To solve this problem, milk powder is often used as a supplement or substitute for breast milk. Furthermore, pigs represent an ideal biomedical model for humans, as their digestive tract structure and length can accurately simulate the process of food delivery in the human digestive tract, additionally, their microbiota can effectively reflect that of the human system performance [19]. To our knowledge, a previous study has compared gut microbial gene expression in 21-day-old neonatal piglets between BF and FF using pyrosequencing-based whole transcriptome shotgun sequencing [20]. However, sequencing technologies and database annotations are now more precise than ten years ago, and the study was conducted on Western pigs. Therefore, we present a comprehensive comparison of gut microbiomes from two different feeding patterns (BF vs. FF) in weaned Rongchang piglets using pair-end sequencing. This study highlights the impact of breastfeeding on the formation of early gut microbiota and provides theoretical support for indigenous Chinese pig production industries, and also provides useful references for human gut microbe research.

## 2. Materials and Methods

### 2.1. Animals

The Rongchang piglets used in this experiment were obtained from the Chongqing Animal Science Academy, Chongqing, China. Twelve healthy female vaginally-delivered piglets (birth weight: 0.96 ± 0.06 kg; age: 1 day old) were generated from healthy, multiparous mothers and allowed to suckle for 48 h postnatally in order to obtain colostrums, and divided into two groups (*n* = 6/group). One group from a single litter was housed with their mothers and exclusively BF. The other group from three separate litters was FF and housed individually in a pen, and reared by an automatic milk feeder containing a special formula designed for piglets. The formula was a milk-based sow milk replacer, which contains ~18% crude protein, ~13% crude fat, ~2% crude fiber, ~8% crude ash, ~9% moisture, ~3% lysine, ~1.5% methionine, ~1% Ca, ~0.8% total P, ~0.5% NaCl, ~40 mg/kg VE, ~100 mg/kg VK_3_, ~8 g/kg Fe and ~3 g/kg Zn (China). The formula and warm water (~40 °C) were brewed at a ratio of 1:6 between the times of 0600 and 2100 h and was offered 15 times daily. All piglets were housed in the same animal facility (temperature: 31.5 ± 2.45 °C; relative humidity: 65.16 ± 3.93) and had *ad libitum* access to water, and none had access to any solid food. To prevent crossover, two groups of piglets were separated by a buffer zone of 5 m. The piglets were weaned at the age of 30 days and weighed in the morning, and then the samples were collected.

### 2.2. Sample Collection

Venous blood (15 mL) of the anterior vena cava was collected without anticoagulant from each fasted (12 h) pig. The tubes with blood samples were kept still for 10 min and then centrifuged at 1800× *g* for 10 min at RT, and then the resultant sera were stored at −80 °C. Fecal samples were collected by sterile gloves and put into sterile cryotubes, immediately frozen in liquid nitrogen, pending transport, on dry ice, to a −80 °C storage facility.

### 2.3. Serum Metabolism Indicators

Serum concentrations of aspartate aminotransferase (AST), alanine aminotransferase (ALT), alkaline phosphatase (ALP), total cholesterol (TC), triglycerides (TG), high-density lipoprotein (HDL), low-density lipoprotein (LDL), albumin (ALB), globulin (GLO), γ-glutamyl transferase (GGT), total protein (TP), creatinine (CREA), glucose (GLU), and uric acid (UA) were determined by using CL-8000 clinical chemical analyzer (Shimadzu, Kyoto, Japan) via standard enzymatic procedures.

### 2.4. DNA Extraction and 16S rRNA Sequencing

Fecal samples were thawed once, and bacterial genomic DNA was extracted using the QIAamp DNA Stool Mini Kit (Qiagen, Hilden, Germany) following the manufacturer’s protocol. The V3-V4 region of the 16S rRNA gene was amplified by PCR using the 319F and 806R primers 5′-ACTCCTACGGGAGGCAGCAG-3′ (forward primer) and 5′-GGACTAC HVGGGTWTCTAAT-3′ (reverse primer), and a sequencing adapter was added to the end of the primers. Then the PCR products were purified, quantified, and homogenized to establish a sequencing library. The constructed library was subjected to library quality inspection, and the qualified library was paired-end sequenced on an Illumina HiSeq 2500 platform (Illumina, San Diego, CA, USA) according to the standard protocols. All samples were included in the same sequencing run and the sequences were deposited in the NCBI’s Sequence Read Archive with the accession number PRJNA743993.

### 2.5. Microbial Community Analysis

To get high-quality clean reads, raw reads were further filtered according to the following rules: (1) removing reads containing <80% of bases with quality (Q-value) > 20; (2) removing reads containing unknown nucleotides, and (3) removing primer and adapter contamination. Paired-end clean reads were merged as raw tags using FLASH (v 1.2.11) with a minimum overlap of 15 bp and mismatch error rates of 10%. QIIME 2 was used to filter the noisy sequence of raw tags to obtain high-quality clean tags. The effective tags were clustered into operational taxonomic units (OTUs) of ≥97% similarity using UPARSE. Representative sequence for each OTU was blasted with the Greengene database (v201305) for species annotation based on the RDP classifier (v2.2) with a confidence threshold of 0.6.

OTU rarefaction curve and rank abundance curves were plotted in QIIME. α-diversity was evaluated by richness (ACE and Chao1) and diversity (Simpson and Shannon) using Mothur (v1.30). The β-diversity was evaluated by unweighted UniFrac distances of principal coordinate analysis (PCoA) using QIIME 2. Taxa enrichment was assessed by linear discriminant analysis (LDA) effect size (LEfSe) with default parameters and logarithmic LDA score threshold of 2. PICRUSt was used to detect the composition of functional genetics of each group by comparing species information and analyzing differences between groups in KEGG Orthologs composition including function.

### 2.6. Statistical Analysis

The data analyses were performed with SPSS 22.0 software (IBM Corporation, Armonk, NY, USA, 2014) using Student’s paired *t*-test. All data are shown as means ± standard deviation. Differences were considered statistically significant when *p* < 0.05 throughout.

## 3. Results

### 3.1. Phenotype Characteristics

**Figure 1:**
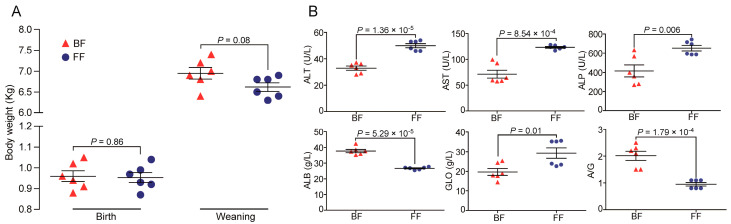
Phenotypic differences: (**A**) Body weights; (**B**) Serum metabolism indicators.

As shown in Figure 1A, there were no significant differences in either birth or weaning body weight between the BF and FF groups (*p* > 0.05), however, the weaning weight of BF piglets tended to be higher than FF ones (*p* = 0.08). Measurement of metabolism indicators in the serum samples revealed that the concentrations of ALT, AST, ALP, ALB, GLO, and A/G were significantly different between the BF and FF piglets (Figure 1B, *p* < 0.05). In contrast, the remaining 10 serum metabolic indicators tested in the BF and FF piglets were not significantly different between the groups (*p* > 0.05, Figure S1). These phenotypic differences between the BF and FF piglets imply the existence of gut microbiota compositional differences.

### 3.2. Microbiota Compositions

**Figure 2:**
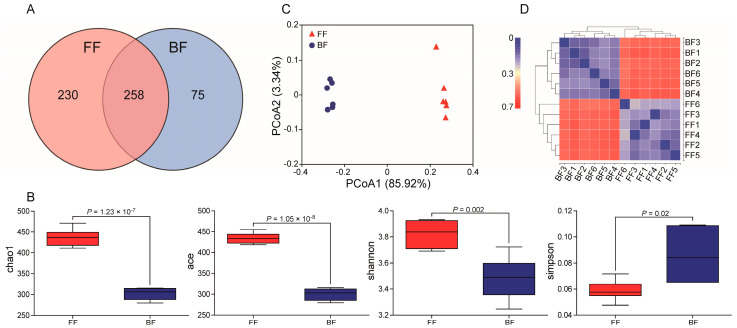
Microbial community structure: (**A**) Venn diagram of operational taxonomic units (OTUs); (**B**) α-diversity; (**C**) Principal coordinate analysis (PCoA) on unweighted UniFrac distances; (**D**) Heat map matrix of β-diversity. The larger the index, the greater difference between samples.

A total of 827,217 raw tags (mean: 68,935; range: 68,376–69,552) were obtained from sequencing, of which 597,697 were clean tags (mean: 49,808; range: 47,777–51,491, Table S1). The species accumulation curve (Figure S2A) and the rarefaction curve (Figure S2B) of the samples combined suggested high-quality sequencing data. Following taxonomic assignment, 488 (range: 386–416) and 333 (range: 260–287) OTUs were obtained from the FF and BF piglets, respectively, and 258 OTUs were shared by both groups (Figure 2A). Furthermore, FF and BF piglets had 230 and 75 unique OTUs, respectively, indicating that piglets exclusively FF had more specific OTUs than the piglets who were fed exclusively with breast milk.

α-diversity analysis showed that there were significant differences in richness and diversity between the FF and BF piglets when comparing Chao1 (*p* = 1.23 × 10^−7^), ACE (*p* = 1.05 × 10^−8^), Shannon (*p* = 0.002) and Simpson (*p* = 0.02). Chao1, ACE, and Shannon in the FF piglets were higher than in the BF piglets; however, Simpson in the FF piglets was lower than observed in the BF piglets (Figure 2B), which suggested that the FF piglets had higher microbiota diversities. A similar trend in microbial diversity was found in the OTU rank curve (Figure S3). In terms of β-diversity, the analysis revealed that the community structures of microbiota were well-clustered by feeding patterns and differed significantly between the groups (*r*^2^ = 0.06, *p* = 0.002) based on the unweighted PCoA plots (Figure 2C, data explained 89.26% of the variation). Furthermore, a clustering matrix heatmap on the basis of β-diversity showed that the biological replicates were similar to each other and all of the individuals could be clearly assigned to a group (Figure 2D), thus suggesting experimental reliability and further highlighting the low variation in gut microbial profiles across different individuals.

### 3.3. Microbial Phyla and Genera

**Figure 3:**
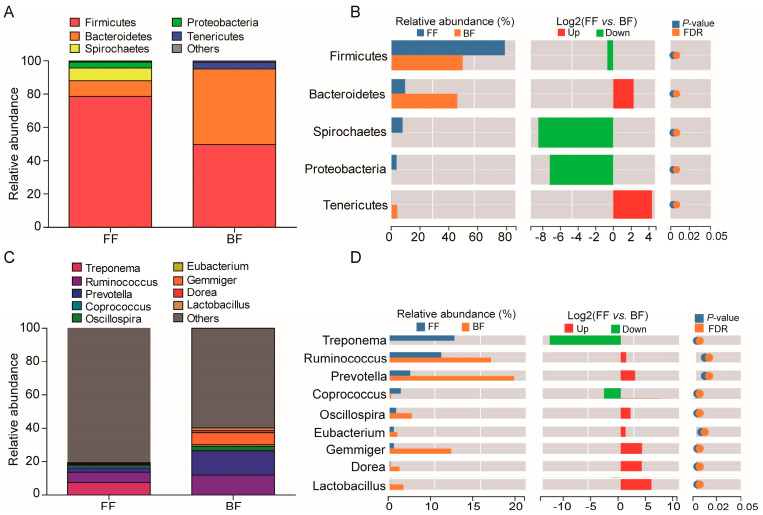
Microbial community composition: (**A**) Composition at the phyla level; (**B**) Relative abundance at the phyla level; (**C**) Composition at the genera level; (**D**) Relative abundance at the genus level.

As shown in Figure 3, five dominant phyla were identified within the two groups, among which Firmicutes and Bacteroidetes constituted the most dominant phyla (FF: 88.08%; BF: 95.13%). In addition, Spirochaetes (7.72%) and Proteobacteria (3.51%) were abundant in the FF piglets, whereas Tenericutes (4.03%) was enriched in the BF piglets (Figure 3A). Metastats-based analysis of the differential abundances between these phyla confirmed that Firmicutes (*p* = 0.005), Spirochaetes (*p* = 0.005), and Proteobacteria (*p* = 0.005) were significantly decreased in BF piglets, whereas Bacteroidetes (*p* = 0.005) and Tenericutes (*p* = 0.005) were significantly increased in the BF piglets (Figure 3B). At the genera level, a total of nine genera were found to be the most abundant between the two groups, and *Ruminococcus*, *Prevotella*, and *Gemmiger* were the most prominent (Figure 3C). Interestingly, all of the nine genera showed distinct changes. The relative abundances of *Ruminococcus* (*p* = 0.005), *Prevotella* (*p* = 0.02), *Oscillospira* (*p* = 0.005), *Eubacterium* (*p* = 0.01), *Gemmiger* (*p* = 0.005), *Dorea* (*p* = 0.005), and *Lactobacillus* (*p* = 0.005) in the BF piglets were significantly higher than FF piglets, whereas *Treponema* (*p* = 0.004) and *Coprococcus* (*p* = 0.005) were significantly lower (Figure 3D). These results indicate that feeding patterns have no effect on microbial community composition at the phyla and genera levels, but can cause disproportional abundances between the BF and FF piglets.

### 3.4. Differences in Microbiota

**Figure 4:**
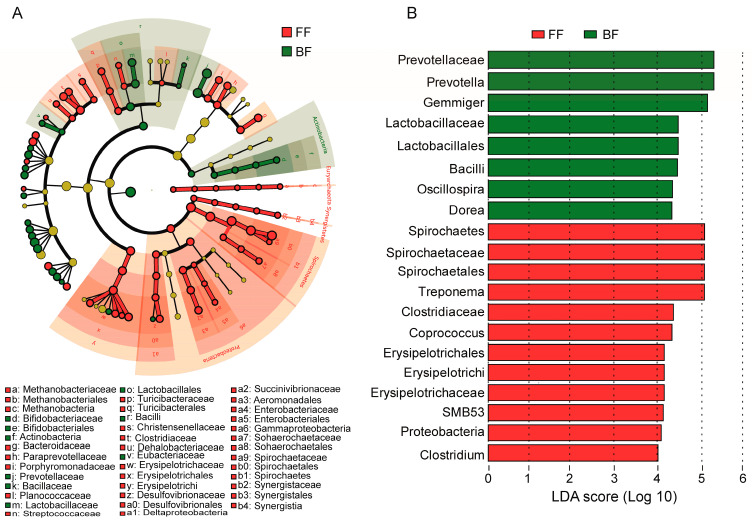
Differential microbiota: (**A**) Discriminant analysis of microbiota enrichment; (**B**) Linear discriminant analysis (LDA) effect size (LEfSe). Only the logarithmic LDA scores > 4 are shown for demonstration and clarity.

LEfSe was performed to further discriminate between the differences observed in microbiota taxa abundance between the BF and FF piglets. As shown in Figure 4A, the phylogenetic composition of the microbiota was noticeably different between the BF and FF piglets. The results showed that a total of 94 microbiota biomarkers at five different taxonomic levels were differentially abundant (Figure 4A: LDA score > 4; Figure S4: 2 < LDA score < 4). In comparison, FF piglets accounted for the majority of the 94 microbiota clades (68.09%). Spirochaetales and Erysipelotrichales were enriched in the FF piglets, whereas Lactobacillales was enriched in the BF piglets (Figure 4B). Furthermore, several discriminant taxa were identified that were not differentially abundant in the conventional analysis, such as *Clostridiaceae* and *SMB53*. It was also noteworthy that some important microbiota were differentially abundant between the BF and FF piglets (Figure S4). For example, *Paraprevotellaceae*, *Gammaproteobacteria*, *Succinivibrionaceae*, *Aeromonadales*, and *Desulfovibrionaceae* were abundant in the FF piglets, whereas BF piglets were associated with the highest relative abundance of *Megasphaera*, Bacillaceae, and Bifidobacteriaceae.

### 3.5. Potential Functional Prediction

**Figure 5:**
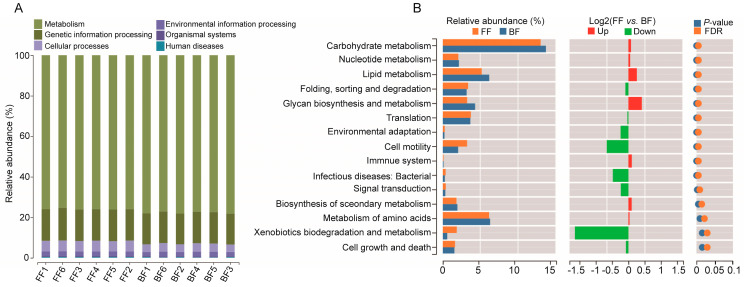
Microbiota functional differences analysis: (**A**) Microbiota function predicted based on the KEGG database (at level I). (**B**) The relative abundance of KEGG metabolic pathways (at level II). Only *p* < 0.05 is shown for demonstration and clarity.

Function prediction analysis showed that >75% of the microbiota were enriched within the metabolism pathway (Figure 5A, BF: 75.3~76.15%, average 75.9%; FF: 77.09~78.23%, average 77.69%), followed by pathways for genetic information processing (BF: average 15.7%; FF: average 15.42%) and cellular processes (BF: average 5.25%; FF: average 3.93%). Further analysis showed that most of the predicted pathways (15 of 21) in the BF piglets were significantly different from those observed in the FF piglets (Figure 5B, *p* < 0.05). These included carbohydrate metabolism, nucleotide metabolism, lipid metabolism, glycan biosynthesis and metabolism, immune system, biosynthesis of secondary metabolism, and amino acid metabolism pathways which were enriched in BF piglets. The FF piglets were abundant in folding, sorting and degradation, translation, environmental adaptation, cell motility, infectious diseases: bacterial, signal transduction, xenobiotics biodegradation and metabolism, and cell growth and death. The results suggested that different feeding patterns had different functions for the microbial community.

### 3.6. Associations between Microbiota and Phenotypes

**Figure 6:**
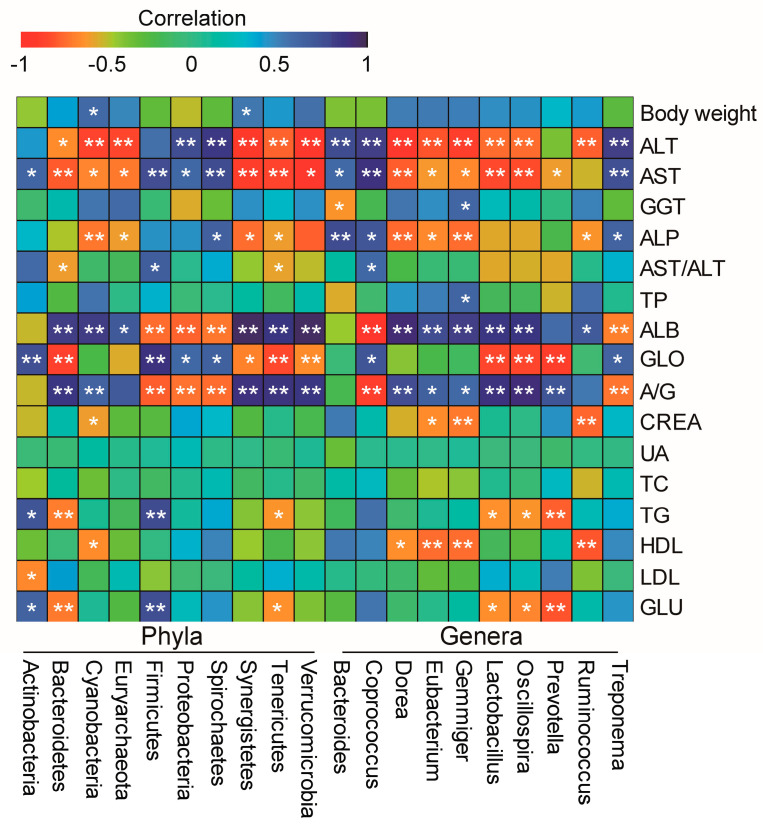
Heatmap diagram representing the correlation between the top 10 abundance of microbiota and phenotypes. * *p* < 0.05, ** *p* < 0.01.

To understand whether the dominant microbiota caused phenotypic differences, the association between the top 10 microbiota and phenotypes was analyzed (Figure 6). As expected, significant differences in ALT, AST, ALP, ALB, GLO, and A/G (Figure 1B) were significantly related to almost all of the top 10 abundance of microbiota at both phyla and genera (*p* < 0.05). Furthermore, except for Euryarchaeota, Proteobacteria, Spirochaetes, Verrucomicrobia, and Treponema, other microbiota were also more or less associated with some of the other phenotypes (*p* < 0.05), including TG, HDL, and GLU.

## 4. Discussion

In the present study, the gut microbiota of BF- and FF-weaned piglets (at 30 days old) were compared and the associations between gut microbiota and feeding patterns were validated. The microbiota was significantly different between the BF- and FF-weaned piglets and many were significantly related to blood metabolism, which suggested that the structure of gut microbiota in the early postnatal period may affect growth traits in pigs through host-microbe interactions. It is noteworthy that some effects of BF and FF on infant gut microbiota are well-known [7,21,22]. However, there are ethical issues in conducting nutritional intervention studies on healthy human infants, therefore more results from experimental animal studies are needed. To our knowledge, our findings are novel and represent the first study establishing a functional linkage between gut microbiota and feeding patterns in indigenous Chinese pigs using paired-end sequencing methodologies.

In humans, the impacts of BF and FF on infant growth rates are well-characterized. Dewey et al. indicated that BF infants are leaner than FF infants at 12 months old [13]. Heinig and colleagues described more rapid decelerations in growth rates in infants BF for more than 12 months compared with those of FF infants after the first three months [23]. These observations were also confirmed when studying infant rhesus macaques, which showed that the overall growth trajectories of FF infants were deemed faster than BF infants [12]. The lower growth rates of BF infants may be attributed to their milk intake self-regulation which could result in lower intake levels than FF infants [24]. Our results showed that no differences were observed in body weight between BF and FF piglets once they had reached 30 days old. It is possible that BF fulfills the needs of the tissues and organs, and allows normal growth in postnatal pigs short-term. Interestingly, the body weight of the BF animals tended to be higher than the FF piglets (*p* = 0.08), which was inconsistent with previous studies [12,13]. This may be caused by differences between species or formula types, or that pigs may have formed different energy and protein utilization abilities from humans during evolution, and the composition and content of piglet formula which lacks bioactive molecules such as oligosaccharides, are not as perfect for human infants. The BF piglets could also receive breast milk when FF piglets fast at night. Studies in humans have shown that rapid weight gain during infancy due to FF can lead to later risks associated with adult obesity, dyslipidemia, and insulin resistance [25,26]. However, pigs are resistant to the spontaneous development of type 2 diabetes mellitus, insulin resistance, and obesity, even after intervention with high-fat, high-fructose, and high-carbohydrate diets [27], which further confirms the different digestion and metabolism processes between pigs and humans. Moreover, it is evident that FF increases basal blood glucose and decreases plasma ketone body concentrations in infants [24]. Similarly, our results also revealed that several serum metabolic indicators were significantly different between the BF and FF piglets, such as FF piglets having higher concentrations of serum ALT, AST, and GLO, which is potentially correlated with health and immunity. Kulkarni et al. [28] showed that the BF infants had less liver damage compared with the FF infants, as indicated by the lower AST and ALT. Saarnen et al. [29] found that serum IgA was increased in FF piglets compared to that of BF breeds.

The number of OTUs, diversity, and richness of the microbiota was higher in FF piglets than in BF piglets, which shows conformity with a previous report indicating that the feeding patterns were strongly associated with richness, diversity, and composition of gut microbiota [7]. Previous studies have reported that gut microbiota diversity in BF was lower than in FF in healthy infants [7,22], and the gut microbiota of FF infants is similar to older children [30]. In humans, a low diversity in gut microbiota during early life appeared to characterize a healthy gut, because specific bacteria are selected for degrading particular oligosaccharides in breast milk [22]. Although the ingredients of commercial formulas are getting increasingly similar to breast milk over time, more of them contain basic nutrients and lack bioactive ingredients. Furthermore, gut microbiota diversity increases with age, indicating a more complex microbial community over time [31]. Compared with this study, the microbial community of BF and FF in 21-day-old piglets were generally similar in terms of the cecal contents [20]. Ma and coauthors indicated that fecal bacterial diversity in healthy infants was lower in BF than in FF individuals at 40 days old, but this increased significantly by the age of six months [22]. These findings suggested that the age of the study subject has different effects on gut microbiota, therefore, different studies cannot simply be compared with each other.

In this study, the main phyla present in all piglets were Firmicutes and Bacteroidetes, which is consistent with similar studies in human infants [7] and piglets [20], suggesting that Firmicutes and Bacteroidetes are commonly important, and numerous within the gut microbiota. These findings conform with another study showing that the majority of taxa detected in human milk belong to Firmicutes and Bacteroidetes [4]. The relative abundance of Firmicutes was higher in FF piglets, whereas Bacteroidetes numbers were higher in the BF piglets, which was in agreement with the report showing that BF monkeys have higher Bacteroidetes levels [12], as did infants [7,22]. However, another study in piglets reported that the Bacteroidetes population was significantly higher in the FF group [20]. This difference may have resulted from the pig breeds forming in different geographical regions, as human studies have shown that one dominated by Bacteroides was more commonly found in Western (American and Western European) subjects and the other which was dominated by Prevotella was more frequently associated with non-Western subjects [32,33]. Another study indicated that the Bacteroidetes is increased following the introduction of solid foods [34] and that this is also associated with diversity and faster maturation of the gut microbiome [35]. Bacteroidetes are related to an animal protein and saturated fat diet, and therefore the relative abundance of Bacteroidetes in gut microbiota is highly susceptible to dietary changes [33]. Moreover, Bacteroides are among several beneficial bacteria in the earlier neonatal phase, which play important and specific functions in the development of the mucosal immune system [36].

The *Ruminococcus*, *Prevotella*, and *Gemmiger* genera were predominant in all of the piglets. This finding was different from previous reports showing that infants were dominated by *Bifidobacterium*, *Enterobacteriaceae*, and *Lactobacillus* [7,22], whilst *Prevotella*, *Blautia*, and *Lactobacillus* were enriched in monkeys [12]. The *Ruminococcus* genus is directly associated with breast milk oligosaccharides [37], which produce both butyrate and a bacteriocin, ruminococcin A, and enable inhibition of the growth of potentially harmful species [38]. The relative abundance of *Prevotella* was in accordance with a previous report showing it was higher in BF piglets than in FF piglets [20]. BF monkeys also had a higher relative abundance of *Prevotella* than FF monkeys [12]. *Prevotella* are critically important for the regulation of immune responses because they contribute towards the production of fermentation enzymes responsible for short-chain fatty acids [39]. *Prevotella* has been presumed to represent consistent underlying microbial communities in humans, making them common biomarkers of diet and lifestyle [33]. There have been numerous studies reporting increases in *Gemmiger*, which is butyricum, often considered to have beneficial effects on health outcomes [40,41,42,43]. In addition, Taylor and colleagues [44] showed that *Gemmiger* was particularly strongly associated with diet, findings that were based on large-scale analysis using population-based studies. Surprisingly, *Treponema* represented the most abundant genus in FF piglets but was nearly absent in BF piglets. It is the etiological agent of swine dysentery, a mucohemorrhagic diarrheal disease in post-weaning pigs. Indeed, the FF piglets in this study had mild diarrhea after weaning. BF has positive benefits for providing sustained protection against enteric pathogens. Specifically, oligosaccharides in breast milk selectively stimulate the propagation of microbes to upregulate genes involved in the metabolism of host glycans to provide protection against diarrheal disease [45].

Based on LEfSe analysis, we also found that Lactobacillales and Bifidobacterium were enriched in the BF piglets, which is consistent with studies comparing BF and FF in infants [7,46], monkeys [12] and piglets [20], suggesting that BF favored the growth of commensal Lactobacillus and Bifidobacteria [47,48]. This may be due to vertical transmission and stimulation from prebiotic oligosaccharides in breast milk. One study has shown that BF newborns carry a more stable and uniform population of oligosaccharides compared with FF newborns [48]. Oligosaccharide supplementation of formula can specifically stimulate the growth of both Bifidobacteria and Lactobacilli and reduce the growth of pathogens [49]. Lactobacillus is a probiotic which showed protective effects in infants, including the reduction of infant colic incidence and reduced use of pain-relieving agents [50]. Studies of infants who received Lactobacillus during their early lives showed that the Lactobacillus resulted in significantly less crying, lowered the presence of *Clostridium histolyticum*, and reduced viral respiratory tract infections [51,52]. In contrast, disease-related pathogens Spirochaetales [53,54] and Erysipelotrichales [55,56] were abundant in the FF piglets. Similarly, the relative abundance of the *Erysipelatoclostridium* genus in the FF infants was higher than in BF infants [7]. In other studies, the foregut microbiomes of the captive-reared Amargosa vole reared on commercial diets, were dominated by Erysipelotrichales [57], and fecal Erysipelotrichales was significantly affected by a high-fat diet in two-month-old male C57BL/6 mice [58]. This evidence suggested that Erysipelotrichales could be considered a potential marker of diet-related changes.

Function prediction analysis showed that most microbiota were enriched in terms of the metabolism pathway. It is well-defined that metabolites from microbiota may play a role in cell-to-cell communication with their host and thus affect host metabolism [59]. Valeriy et al. reported that the enzymes related to carbohydrate and protein metabolism were enriched in BF and FF piglets, while the abundance of enzymes related to amino acid metabolism was different [20]. In vitro, transketolase expression was upregulated after exposure to breast milk [60]. Transketolase is an enzyme involved in central carbohydrate metabolism that has been shown to link carbon availability [61]. The amino acids metabolism pathway enrichment observed in BF piglets was consistent with the previous studies showing that BF piglets had enriched arginine relative to FF [20] and that amino acid synthesis pathways were increased in the microbiota of BF infants [62]. Amino acids in breast milk are considered to be an essential source related to fast-growing BF infants and play a role in promoting the digestion and absorption of other nutrients. Interestingly, an abnormally low serum concentration of arginine, a precursor for nitric oxide production, confers an increased risk for necrotizing enterocolitis, and it is widely accepted that the incidence of necrotizing enterocolitis is lower in infants receiving breast milk [63]. Moreover, our findings showed that *Lactobacillus* was significantly positively related to ALB and A/G, whereas it was significantly negatively related to ALT, AST, GLO, TG, and GLU. Dietary supplementation with *Lactobacillus plantarum* reduced the contents of TG, ALT, and AST in the serum of obese mice [64] and increased the serum A/G in rabbits [65]. *Lactobacillus* also relieved varying degrees of liver pathological changes in mice, and significantly decreased the expression levels of both AST and ALT in serum [66]. Firmicutes was significantly positively associated with AST, GLO, TG, and GLU, whereas it was significantly negatively associated with ALB and A/G. Yue et al. previously reported that obese rats have a higher proportion of Firmicutes in the gut and the serum TG content was significantly increased [67]. However, the opposite result was seen in the findings by Moreno-Navarrete [68]. These findings confirmed that the phenotypic differences are directly and indirectly affected by gut microbiota composition or activity [69,70,71].

## 5. Conclusions

The present study validated the differences in gut microbiota composition in these piglet groups according to different feeding patterns. Gut microbiota diversity was lower in the weaned BF piglets than in the FF ones. The BF piglets were enriched with Lactobacillales and Spirochaetales, whereas Erysipelotrichales was enriched with the FF piglets. The carbohydrate and amino acid metabolism of the BF piglets was significantly different from those of the FF piglets. These results partly interpreted the importance of BF from the perspective of gut microbiota, and have a certain guiding role in the feeding patterns employed in indigenous Chinese pigs. However, long-term studies are needed to determine the effects of different early feeding patterns on the growth and health of adult pigs.

## Data Availability

The datasets used and/or analyzed during the current study are available from the corresponding author upon reasonable request.

## Supplementary Materials

The following supporting information can be downloaded at: https://www.mdpi.com/article/10.3390/genes14010049/s1, Table S1: Statistics of sequencing data; Figure S1: Serum metabolism indicators; Figure S2: Evaluate the quality of sequencing data. (A) Species accumulation curve. (B) Rarefaction curve; Figure S3: Operational taxonomic unit (OUT) rank curve; Figure S4: Linear discriminant analysis (LDA) effect size (LEfSe). Only the logarithmic 2 < LDA score < 4 is shown for demonstration and clarity.
