# Supplementary material for: Distinct Gut Microbiome Induced by Different Feeding Regimes in Weaned Piglets
Source: Genes (Basel). 2022 Dec 23;14(1):49. doi: 10.3390/genes14010049 (PMC9858795; doi:10.3390/genes14010049)
Supplement: Supplementary file 1 [file genes-14-00049-s001.zip › Table S1.pdf]

Table S1. Statistics of sequencing data

Sample	Raw data (Mb)	Clean data (Mb)	Data utilization ratio (%)	Raw reads	Clean reads	Read utilization ratio (%)	Connect tags	Connect ratio (%)	Clean tags	OTUs
FF1	39.94	37.5	93.9	72063	69221	96.06	69104	99.83	49495	386
FF2	39.82	37.5	94.16	72003	69190	96.09	69050	99.8	50948	401
FF3	39.84	37.5	94.13	72151	69411	96.2	69296	99.83	50319	413
FF4	39.77	37.5	94.29	72280	69583	96.27	69445	99.8	51491	416
FF5	39.65	37.5	94.57	72227	69682	96.48	69552	99.81	51365	404
FF6	39.81	37.5	94.2	71588	68850	96.18	68743	99.84	49087	386
BF1	39.71	37.5	94.44	71517	68904	96.35	68696	99.7	47777	285
BF2	40.15	37.5	93.39	71792	68560	95.5	68376	99.73	48772	287
BF3	40.24	37.5	93.2	72088	68784	95.42	68602	99.74	48005	287
BF4	40.05	37.5	93.63	71881	68740	95.63	68589	99.78	49136	274
BF5	40.1	37.5	93.53	72085	68891	95.57	68737	99.78	50702	260
BF6	40.13	37.5	93.46	72383	69153	95.54	69027	99.82	50600	281
