# Supplementary figures and images for: Distinct Gut Microbiome Induced by Different Feeding Regimes in Weaned Piglets
Source: Genes (Basel). 2022 Dec 23;14(1):49. doi: 10.3390/genes14010049 (PMC9858795; doi:10.3390/genes14010049)

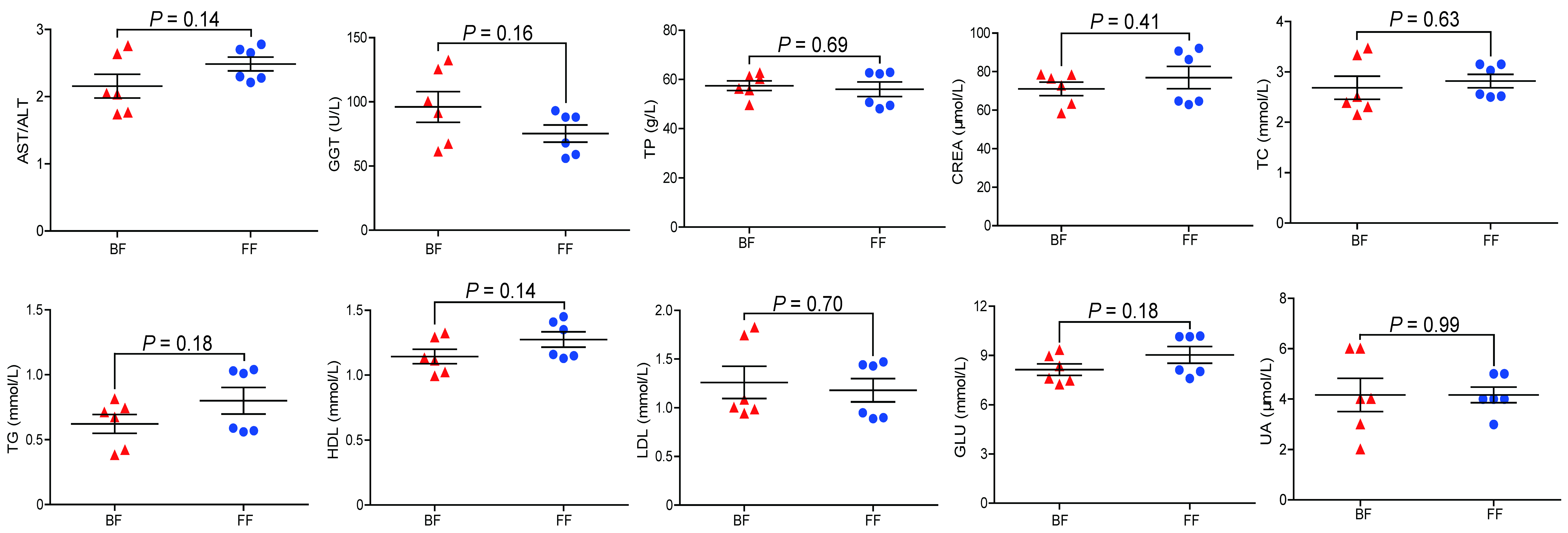

Supplement: Supplementary file 1 [file genes-14-00049-s001.zip › Figure S1.tiff]

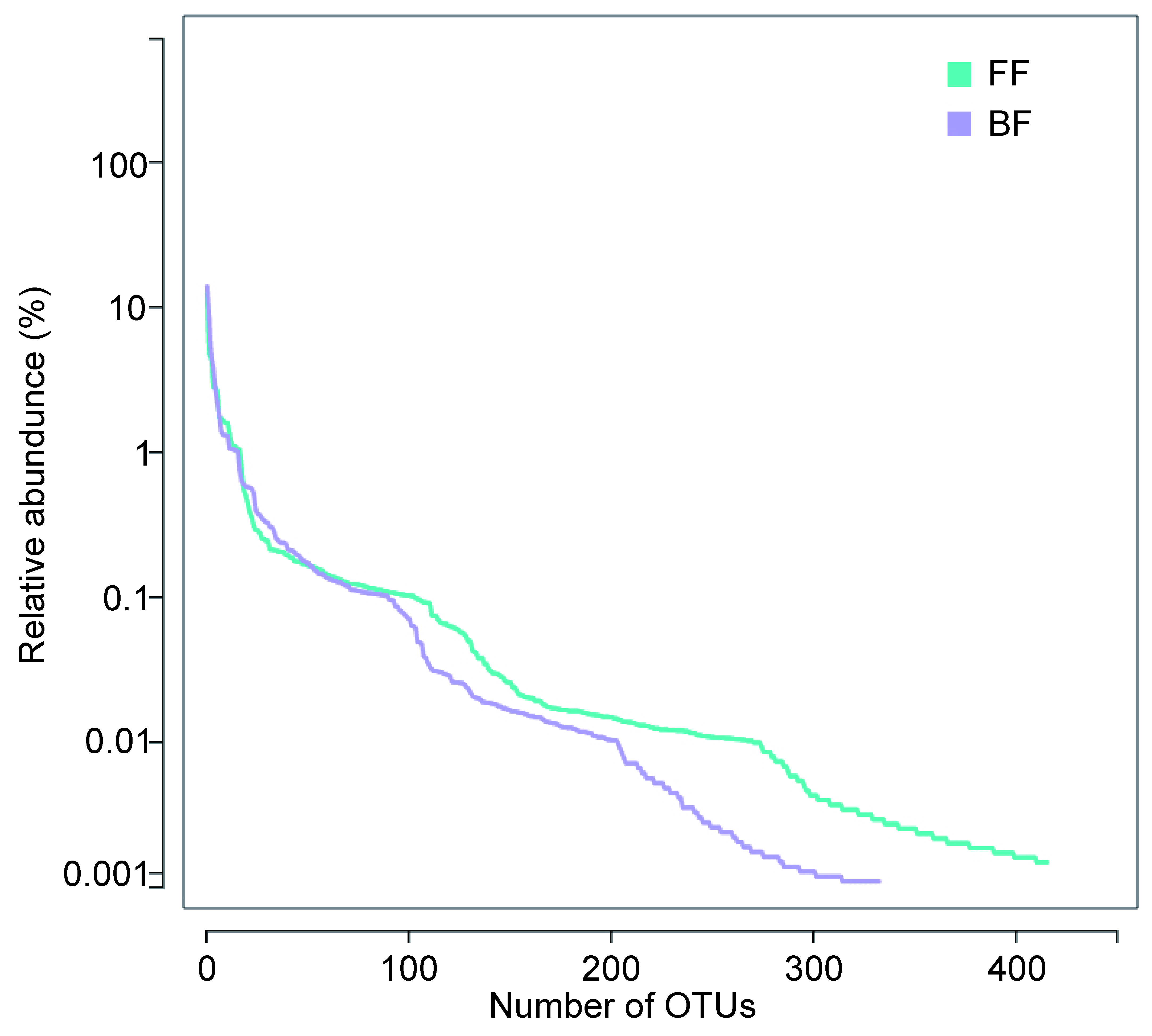

Supplement: Supplementary file 1 [file genes-14-00049-s001.zip › Figure S3.tiff]

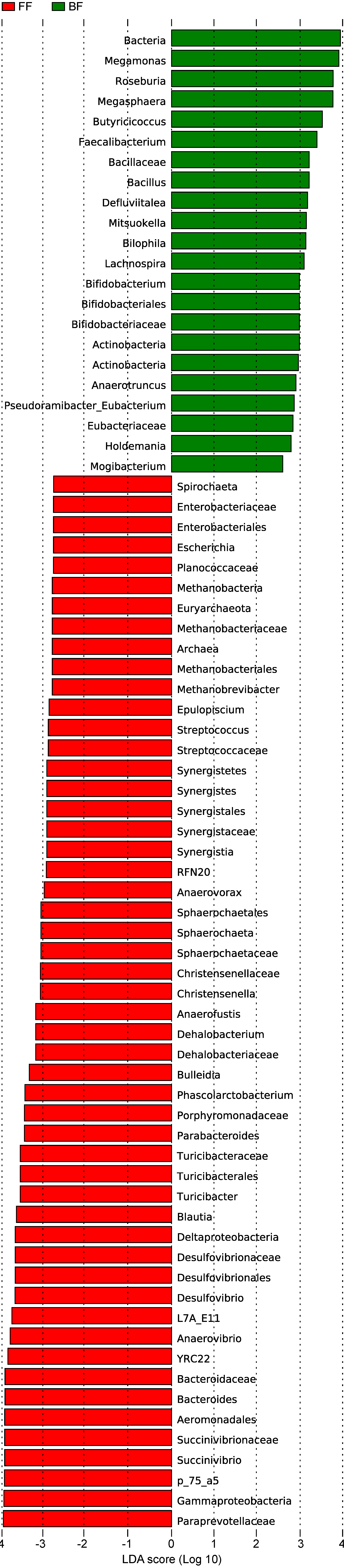

Supplement: Supplementary file 1 [file genes-14-00049-s001.zip › Figure S4.tiff]
